# Associations between lncRNA* MEG3* polymorphisms and neuroblastoma risk in Chinese children

**DOI:** 10.18632/aging.101406

**Published:** 2018-03-27

**Authors:** Zhen-Jian Zhuo, Ruizhong Zhang, Jiao Zhang, Jinhong Zhu, Tianyou Yang, Yan Zou, Jing He, Huimin Xia

**Affiliations:** 1 Department of Pediatric Surgery, Guangzhou Institute of Pediatrics, Guangzhou Women and Children’s Medical Center, Guangzhou Medical University, Guangzhou 510623, Guangdong, China; 2 School of Chinese Medicine, Faculty of Medicine, The Chinese University of Hong Kong, Hong Kong 999077, China; 3 Department of Pediatric Surgery, the First Affiliated Hospital of Zhengzhou University, Zhengzhou 450052, Henan, China; 4 Department of Clinical Laboratory, Molecular Epidemiology Laboratory, Harbin Medical University Cancer Hospital, Harbin 150040, Heilongjiang, China; 5 Sun Yat-sen University Cancer Center, State Key Laboratory of Oncology in South China, Department of Experimental Research, Collaborative Innovation Center for Cancer Medicine, Guangzhou 510060, Guangdong, China; * Equal contribution

**Keywords:** *MEG3*, polymorphism, neuroblastoma, susceptibility

## Abstract

Neuroblastoma is the third most common childhood cancer after leukemias and cancer of the central nervous system. Long noncoding RNA *MEG3* polymorphisms have been shown to confer cancer susceptibility; however, their roles in the genetic predisposition to neuroblastoma remain unclarified. To answer this question, we genotyped two *MEG3* polymorphisms, rs7158663 G>A and rs4081134 G>A, in 392 neuroblastoma children and 783 controls by TaqMan method. The results showed that neither single locus nor the combination analysis supported an association between *MEG3* polymorphism and neuroblastoma risk. Interestingly, we found that subjects carrying rs4081134 AG/AA genotypes significantly tended to develop neuroblastoma among subgroups with age >18 month (adjusted OR=1.36, 95% CI=1.01-1.84) and clinical stage III+IV disease (adjusted OR=1.47, 95% CI=1.08-1.99), when compared with reference group. In the combined analysis of *MEG3* polymorphisms, we found that carriers of 2 risk genotypes were more likely to have higher risk of developing neuroblastoma than those with 0-1 risk genotype among children more than 18 months of age (adjusted OR=1.36, 95% CI=1.01-1.84, *P*=0.042), and with clinical stages III+IV disease (adjusted OR=1.47, 95% CI=1.08-2.00, *P*=0.014). Our data suggest *MEG3* as a weak-effect neuroblastoma susceptibility gene. Well-designed studies with large sample studies are needed to further validate this finding.

## Introduction

Neuroblastoma is a heterogeneous tumor rising from neural crest progenitor cells. It is the most common solid neoplasm in children, accounting for nearly 10% of all childhood cancers [1]. Neuroblastoma is characterized by broadly clinical presentation. Some bearing favorable tumors have spontaneous regression without chemotherapy; others have metastatic disease resistant to even intense treatment [2,3]. Survival rates of patients with the most aggressive form of neuroblastoma are less than 40%, even after receiving intensive therapy [1,4,5].

Neuroblastoma can be classified into familial and sporadic types, according to the presence or absence of germline mutations. Familial neuroblastoma is rare, and accounts for about 1-2% of all cases [6]. Most of the familial neuroblastoma are related to the recurrent mutations in *PHOX2B* [7,8] or *ALK* gene [9,10]. However, the genetic bases of sporadic neuroblastoma, remains largely unknown. Previous studies have suggested that several environmental factors such as dwelling condition, maternal medication use, and childhood infections are potential risk factors for sporadic neuroblastoma [11,12], but direct link is lacking. Growing evidence suggests that genetic variants can modify the risk of neuroblastoma [13–18]. For example, common genetic variants of *NEFL* and *CNKN1B* are associated with neuroblastoma susceptibility [19,20].

Long non-coding RNAs (lncRNAs) are a class of non-coding transcripts with more than 200 nucleotides in length [21]. A plethora of studies have revealed that lncRNAs are implicated in tumorigenesis [22,23]. They can regulate pathophysiological activities of cells in the epigenetic, transcriptional, or posttranscriptional levels [24,25]. So far, numerous polymorphisms in the lncRNAs have been identified to be associated with cancer risk. For example, a genome-wide association study (GWAS) by Guo et al. identified 45 candidate lncRNAs associated with prostate cancer susceptibility [26]. Yang et al. first reported that lncRNA *H19* genetic variants may contribute to gastric cancer risk [27]. Notable, our previous study also revealed the association between *LINC00673* rs11655237 C>T polymorphism and neuroblastoma susceptibility [28].

The lncRNA *MEG3* is a tumor suppressor. It has been involved in several types of cancer, including bladder cancer [29], gastric cancer [30], hepatocellular carcinoma [31], and lung cancer [32]. Accumulating evidence has suggested that genetic variants in the *MEG3* gene predispose to cancer. However, the impacts of *MEG3* polymorphisms in neuroblastoma risk remain unclear. Therefore, we conducted a gene-based association analysis of *MEG3* polymorphisms and neuroblastoma risk.

## RESULTS

### *MEG3* polymorphisms and neuroblastoma susceptibility

In total, 393 cases and 812 controls were included in the current study (Supplementary Table 1), of which, 392 cases and 783 controls were successfully genotyped. Both of polymorphisms (rs7158663 G>A and rs4081134 G>A) were in accordance with Hardy-Weinberg equilibrium (HWE) in the control subjects (rs7158663: HWE=0.725, and rs4081134: HWE=0.762). However, neither of the two *MEG3* polymorphisms was associated with neuroblastoma susceptibility, no matter adjusted for age and gender or not. We next evaluated the effects of combined risk genotypes on neuroblastoma susceptibility. Similarly, no significant association was found (Table 1). Null associations between *MEG3* polymorphisms and neuroblastoma susceptibility were also observed for Guangdong and Henan subjects, individually (Supplementary Table 2).

**Table 1:** Associations between *MEG3* polymorphisms and neuroblastoma susceptibility.

Genotype	Cases (N=392)	Controls (N=783)	*P* ^a^	Crude OR (95% CI)	*P*	Adjusted OR (95% CI) ^b^	*P* ^b^
rs7158663 (HWE=0.725)							
GG	233 (59.44)	433 (55.30)		1.00		1.00	
AG	141 (35.97)	296 (37.80)		0.89 (0.69-1.14)	0.351	0.89 (0.69-1.15)	0.354
AA	18 (4.59)	54 (6.90)		0.62 (0.36-1.08)	0.092	0.62 (0.35-1.07)	0.088
Additive			0.193	0.84 (0.69-1.03)	0.088	0.84 (0.68-1.03)	0.086
Dominant	159 (40.56)	350 (44.70)	0.177	0.84 (0.66-1.08)	0.177	0.84 (0.66-1.08)	0.177
Recessive	374 (95.41)	729 (93.10)	0.120	0.65 (0.38-1.12)	0.123	0.65 (0.37-1.12)	0.117
rs4081134 (HWE=0.762)							
GG	200 (51.02)	443 (56.58)		1.00		1.00	
AG	165 (42.09)	294 (37.55)		1.24 (0.97-1.60)	0.092	1.25 (0.97-1.60)	0.090
AA	27 (6.89)	46 (5.87)		1.30 (0.79-2.15)	0.307	1.31 (0.79-2.17)	0.297
Additive			0.193	1.19 (0.98-1.45)	0.083	1.19 (0.98-1.45)	0.079
Dominant	192 (48.98)	340 (43.42)	0.071	1.25 (0.98-1.60)	0.071	1.25 (0.98-1.60)	0.069
Recessive	365 (93.11)	737 (94.13)	0.498	1.19 (0.73-1.94)	0.498	1.19 (0.73-1.95)	0.486
Combine risk genotypes							
0	18 (4.59)	53 (6.77)		1.00		1.00	
1	182 (46.43)	391 (49.94)		1.37 (0.78-2.41)	0.273	1.38 (0.79-2.43)	0.262
2	192 (48.98)	339 (43.30)	0.102	1.67 (0.95-2.93)	0.075	1.68 (0.96-2.96)	0.071
0-1	200 (51.02)	444 (56.70)		1.00		1.00	
2	192 (48.98)	339 (43.30)	0.065	1.26 (0.99-1.60)	0.065	1.26 (0.99-1.61)	0.063

OR, odds ratio; CI, confidence interval.

^a^ *χ^2^* test for genotype distributions between neuroblastoma cases and cancer-free controls.

^b^ Adjusted for age and gender.

### Stratification analysis

Stratification analysis by age, gender, tumor sites of origin and clinical stages was further performed (Table 2). No significant association was identified for rs7158663 G>A and neuroblastoma susceptibility. Interestingly, we found that subjects carrying rs4081134 AG/AA genotypes were at significantly increased risk of developing neuroblastoma among children older than 18 months of age [adjusted odds ratio (OR)=1.36, 95% confidence interval (CI)=1.01-1.84] and those with clinical stage III+IV disease (adjusted OR=1.47, 95% CI=1.08-1.99), when compared with reference group. In addition, combined analysis indicated that the presence of 2 risk genotypes collectively increased neuroblastoma risk in the children >18 months of age (adjusted OR=1.36, 95% CI=1.01-1.84, *P*=0.042), and subgroup with III+IV clinical stages (adjusted OR=1.47, 95% CI=1.08-2.00, *P*=0.014). We further performed stratification analysis for subjects from Guangdong (Supplementary Table 3) and Henan province, separately (Supplementary Table 4). The results showed that 2 risk genotypes increased neuroblastoma risk in subjects with III+IV clinical stage disease from Guangdong province (adjusted OR=1.58, 95% CI=1.08-2.31, *P*=0.019).

**Table 2:** Stratification analysis of *MEG3* polymorphisms with neuroblastoma susceptibility.

Variables	rs7158663 (cases/controls)		AOR (95% CI) ^a^	*P* ^a^	rs4081134 (cases/controls)		AOR (95% CI) ^a^	*P* ^a^	Risk genotypes (cases/controls)		AOR (95% CI) ^a^	*P* ^a^
	GG	AG/AA			GG	AG/AA			0-1	2		
Age, month												
≤18	74/161	51/132	0.84 (0.55-1.29)	0.427	64/156	61/137	1.09 (0.72-1.65)	0.696	64/157	61/136	1.10 (0.72-1.68)	0.650
>18	159/272	108/218	0.85 (0.63-1.15)	0.280	136/287	131/203	**1.36 (1.01-1.84)**	**0.042**	136/287	131/203	**1.36 (1.01-1.84)**	**0.042**
Gender												
Females	101/181	67/147	0.82 (0.56-1.19)	0.297	87/192	81/136	1.31 (0.90-1.91)	0.155	87/192	81/136	1.31 (0.90-1.91)	0.155
Males	132/252	92/203	0.87 (0.63-1.20)	0.380	113/251	111/204	1.21 (0.88-1.67)	0.243	113/252	111/203	1.22 (0.89-1.68)	0.223
Sites of origin												
Adrenal gland	92/433	61/350	0.83 (0.58-1.18)	0.286	80/443	73/340	1.20 (0.84-1.69)	0.318	80/444	73/339	1.20 (0.85-1.70)	0.306
Retroperitoneal	50/433	36/350	0.89 (0.57-1.40)	0.610	43/443	43/340	1.29 (0.82-2.01)	0.269	43/444	43/339	1.29 (0.83-2.02)	0.258
Mediastinum	63/433	46/350	0.91 (0.60-1.36)	0.634	58/443	51/340	1.15 (0.77-1.72)	0.493	58/444	51/339	1.16 (0.77-1.73)	0.478
Others	21/433	15/350	0.88 (0.45-1.73)	0.712	17/443	19/340	1.45 (0.74-2.83)	0.281	17/444	19/339	1.46 (0.75-2.84)	0.273
Clinical stages												
I+II+4s	95/433	67/350	0.87 (0.62-1.23)	0.439	93/443	69/340	0.97 (0.69-1.37)	0.869	93/444	69/339	0.98 (0.69-1.38)	0.892
III+IV	128/433	82/350	0.79 (0.58-1.09)	0.148	99/443	111/340	**1.47 (1.08-1.99)**	**0.015**	99/444	111/339	**1.47 (1.08-2.00)**	**0.014**

AOR, adjusted odds ratio; CI, confidence interval.

^a^ Adjusted for age and gender, omitting the corresponding stratification factor.

## DISCUSSION

To determine the association of the *MEG3* polymorphisms with neuroblastoma risk, we conducted this hospital-based case-control study in Chinese children. Our study provides evidence of the effects of *MEG3* polymorphisms on neuroblastoma susceptibility. Neither of the rs7158663 G>A and rs4081134 G>A significantly modifies neuroblastoma risk. Notably, subjects with rs4081134 AG/AA genotypes were more likely to develop neuroblastoma among subgroup with age >18 month and clinical stage III+IV disease.

LncRNA *MEG3* is located on chromosome 14q32.3. It is the first lncRNA identified as a tumor suppressor, preventing cancer initiation and development [33]. Sun et al. showed that knockdown of *MEG3* expression by siRNA could promote gastric cancer proliferation *in vitro* and decreased expression level of *MEG3* was related to poor prognosis in gastric cancer [34]. A study by Braconi et al. showed that ectopic expression of *MEG3* induced apoptosis in hepatocellular cancer PRC/PRF/5 cells [31]. Another study indicated that a rs116907618 polymorphism in *MEG3* did not significantly affect platinum-based chemotherapy response in lung cancer patients [35]. Recently, Zhou et al. found that lncRNA *MEG3* downregulation contributes to nickel malignant transformation of human bronchial epithelial cells via modulating PHLPP1 transcription and HIF-1α translation [36]. These findings intrigued us to investigate whether *MEG3* might be also involved in neuroblast malignant transformation. The associations between *MEG3* polymorphisms and cancer risks have been also investigated. In a case-control study including 518 cases and 527 controls, Cao et al. genotyped five tagged single nucleotide polymorphisms (tagSNPs) in the *MEG3* (rs3087918, rs11160608, rs4081134, rs10144253, and rs7158663) to investigate their role in colorectal cancer risk [37]. They observed that *MEG3* rs7158663, but not other polymorphisms, was associated with colorectal cancer risk.

Herein, we are the first group to explore the association between *MEG3* polymorphisms and neuroblastoma susceptibility in Chinese children. The results showed that either the *MEG3* polymorphism alone or in combination did not confer neuroblastoma susceptibility. However, another case-control study by Cao et al. [37] demonstrated that *MEG3* rs7158663 AA genotype significantly increased colorectal cancer risk, compared with GG genotype in Chinese population. Their also failed to identify a significant association between rs4081134 and colorectal cancer risk. Polymorphisms may exert diverse genetic effects on cancer susceptibility, depending on different cancer types, geographical regions, and ethnicities. In the stratified analysis, we found that subjects carrying rs4081134 AG/AA genotype significantly tended to develop neuroblastoma among subgroup older than 18 month of age and those with clinical stage III+IV disease, when compared with reference group. It should be noted that this positive association in subgroups might be a chance finding and a result of limited statistical power caused by relatively small sample size.

Though it is the first study performed on the association of interest, limitations accompany. The primary weakness of this study is the relative small sample size. This weakness may impair the strength of the statistical power, especially for the stratification analysis. Second, we only investigated two polymorphisms in the *MEG3* gene. More potentially functional polymorphisms in the *MEG3* are needed to be studied. Third, the results obtained from Chinese children cannot be directly extrapolated to other populations. Finally, only genetic factors were considered in this study, since environmental factors that may influence neuroblastoma risk were not available.

In summary, the present data indicate that *MEG3* polymorphisms have low penetrant effects on neuroblastoma risk. Well-designed case-control studies with larger samples are needed to confirm these findings. Moreover, *in vitro* and *in vivo* functional analysis is warranted to reveal the mechanism how the genetic polymorphisms in *MEG3* affect the neuroblastoma risk.

## MATERIALS AND METHODS

### Study subjects

A total of 393 cases with neuroblastoma and 812 healthy controls of Chinese origin were enrolled for the current study (Supplementary Table 1). The detailed information of these subjects was described in our former studies [28,38,39]. Informed consent was obtained from each participant or their guardian before the research. The study protocols received approval from the Institutional Review Board of Guangzhou Women and Children’s Medical Center, and The First Affiliated Hospital of Zhengzhou University.

### Polymorphism selection and genotyping

In brief, we searched the potentially functional candidate SNPs located in the 5’- flanking region, 5’ untranslated region, 3’ untranslated region, and exon of *MEG3* gene. Two polymorphisms (rs7158663 G>A and rs4081134 G>A) were selected for analysis. Both the two SNPs are located in transcription factor binding sites (TFBS). There is no significant linkage disequilibrium (R^2^<0.8) between these two SNPs in the *MEG3* gene (R^2^=0.08 between rs7158663 and rs4081134) (Supplemental Figure 1). The genomic DNA was firstly extracted from peripheral blood donated by subjects using TIANamp Blood DNA Kit (TianGen Biotech Co. Ltd., Beijing, China). Then the DNA samples were further genotyped on a standard commercial TaqMan real-time PCR [40–43]. More details on genotyping and quality control analyses were reported elsewhere [44–46]. To verify results, 10% of the samples was chosen to a second run. All duplicate sets had a concordance rate of 100%.

### Statistical analysis

Tests for deviation from HWE of the selected polymorphisms in controls were performed by good-of-fit χ^2^ test. Then the two-sided χ^2^ test was adopted to measure the differences in the demographic variables and genotypic frequencies between all cases and controls. ORs and 95% CIs calculated from logistic regression analysis were used to assess the strength of association between *MEG3* polymorphisms and neuroblastoma risk. We used version 9.4 SAS software (SAS Institute, Cary, NC) to conduct all statistical analyses. All the *P* values were two sided, and *P* values less than 0.05 were considered as significant.

## Supplementary Material

Supplementary File
