# Supplementary material for: Associations between lncRNA MEG3 polymorphisms and neuroblastoma risk in Chinese children
Source: Aging (Albany NY). 2018 Mar 27;10(3):481–91. doi: 10.18632/aging.101406 (PMC5892699; doi:10.18632/aging.101406)
Supplement: Supplementary File [file aging-10-101406-s001.pdf]

SUPPLEMENTARY MATERIAL

Supplementary Table 1. Frequency distribution of selected characteristics in neuroblastoma cases and cancer-free controls.

Variables	Guangdong province					Henan province				
	Cases (n=275)		Controls (n=531)		P [a]	Cases (n=118)		Controls (n=281)		P [a]
	No.	%	No.	%		No.	%	No.	%	
Age range, month	0-132		0.07-156		0.229	0-131.1		0.1-144.0		0.484
Mean ± SD	31.50±25.43		29.73±24.86			46.24±29.98		44.97±33.23		
<12	70	25.45	145	27.31		9	7.63	32	11.39	
12-60	177	64.36	313	58.95		76	64.41	179	63.70	
>60	28	10.18	73	13.75		33	27.97	70	24.91	
Gender					0.510					0.196
Female	114	41.45	233	43.88		54	45.76	109	38.79	
Male	161	58.55	298	56.12		64	54.24	172	61.21	
Clinical stages										
I	54	19.64				15	12.71			
II	62	22.55				31	26.27			
III	49	17.82				19	16.10			
IV	94	34.18				49	41.53			
4s	8	2.91				3	2.54			
NA	8	2.91				1	0.85			
Sites of origin										
Adrenal gland	64	23.27				89	75.42			
Retroperitoneal region	87	31.64				/	/			
Mediastinum	90	32.73				19	16.10			
Other region	26	9.45				10	8.47			
NA	8	2.91				/	/			

SD, standard deviation; NA, not available.

[a] Two-sided χ^2 test for distributions between neuroblastoma cases and cancer-free controls.

Supplementary Table 2. Associations between *MEG3* polymorphisms and neuroblastoma susceptibility for Guangdong and Henan subjects.

Genotype	Guangdong province				Henan province			
	Cases (n=274)	Controls (n=502)	AOR (95% CI) [a]	P [a]	Cases (n=118)	Controls (n=281)	AOR (95% CI) [a]	*P* [a]
rs7158663 (HWE=0.799)					IWE=0.957			
GG	168 (61.31)	294 (58.57)	1.00		65(55.08)	139 (49.47)	1.00	
AG	97 (35.40)	179 (35.66)	0.95 (0.69-1.29)	0.723	44 (37.29)	117 (41.64)	0.79 (0.50-1.25)	0.322
AA	9 (3.28)	29 (5.78)	0.54 (0.25-1.17)	0.117	9 (7.63)	25 (8.90)	0.72 (0.32-1.65)	0.442
Additive			0.86 (0.66-1.10)	0.227			0.83 (0.59-1.16)	0.272
Dominant	106 (38.69)	208 (41.43)	0.89 (0.66-1.20)	0.442	53 (44.92)	142 (50.53)	0.78 (0.51-1.21)	0.266
Recessive	265 (96.72)	473 (94.22)	0.55 (0.26-1.18)	0.126	109 (92.37)	256 (91.10)	0.80 (0.36-1.78)	0.587
rs4081134 (HWE=0.208)					HWE=0.224			
GG	131 (47.81)	265 (52.79)	1.00		69 (58.47)	178 (63.35)	1.00	
AG	124 (45.26)	207 (41.24)	1.21 (0.89-1.64)	0.223	41 (34.75)	87 (30.96)	1.20 (0.75-1.91)	0.442
AA	19 (6.93)	30 (5.98)	1.27 (0.69-2.35)	0.441	8 (6.78)	16 (5.69)	1.30 (0.53-3.18)	0.568
Additive			1.17 (0.92-1.49)	0.205			1.17 (0.82-1.66)	0.386
Dominant	143 (52.19)	237 (47.21)	1.22 (0.91-1.64)	0.191	49 (41.53)	103 (36.65)	1.22 (0.78-1.89)	0.386
Recessive	255 (93.07)	472 (94.02)	1.16 (0.64-2.11)	0.617	110 (93.22)	265 (94.31)	1.22 (0.51-2.93)	0.661
Combine risk genotypes								
0	9 (3.28)	28 (5.58)	1.00		9 (7.63)	25 (8.90)	1.00	
1	122 (44.53)	238 (47.41)	1.61 (0.73-3.52)	0.235	60 (50.85)	153 (54.45)	1.16 (0.51-2.64)	0.730
2	143 (52.19)	236 (47.01)	1.89 (0.87-4.13)	0.109	49 (41.53)	103 (36.65)	1.38 (0.60-3.19)	0.454
0-1	131 (47.81)	266 (52.99)	1.00		69 (58.47)	178 (63.35)	1.00	
2	143 (52.19)	236 (47.01)	1.23 (0.91-1.65)	0.174	49 (41.53)	103 (36.65)	1.22 (0.78-1.89)	0.386

AOR, Adjusted odds ratio; CI, confidence interval.
[a] Adjusted for age and gender.

Supplementary Table 3. Stratification analysis of *MEG3* polymorphisms with neuroblastoma susceptibility (Guangdong province).

Variables	rs7158663 (cases/controls)		AOR (95% CI) [a]	*P* [a]	rs4081134 (cases/controls)		AOR (95% CI) [a]	*P* [a]	Risk genotypes (cases/controls)		AOR (95% CI) [a]	*P* [a]
	GG	AG/AA			GG	AG/AA			0-1	2		
Age, month												
≤18	63/129	39/92	0.86 (0.53-1.40)	0.551	49/112	53/109	1.11 (0.69-1.77)	0.672	49/113	53/108	1.13 (0.70-1.81)	0.617
>18	105/165	67/116	0.91 (0.62-1.34)	0.619	82/153	90/128	1.31 (0.90-1.92)	0.165	82/153	90/128	1.31 (0.90-1.92)	0.165
Gender												
Females	73/133	41/86	0.87 (0.55-1.39)	0.563	57/123	57/96	1.28 (0.81-2.02)	0.284	57/123	57/96	1.28 (0.81-2.02)	0.284
Males	95/161	65/122	0.90 (0.61-1.33)	0.593	74/142	86/141	1.17 (0.80-1.73)	0.421	74/143	86/140	1.19 (0.81-1.75)	0.383
Sites of origin												
Adrenal gland	39/294	25/208	0.92 (0.54-1.57)	0.749	29/265	35/237	1.35 (0.80-2.29)	0.265	29/266	35/236	1.36 (0.80-2.30)	0.255
Retroperitoneal	50/294	36/208	1.01 (0.63-1.61)	0.972	43/265	43/237	1.10 (0.70-1.75)	0.673	43/266	43/236	1.11 (0.70-1.76)	0.648
Mediastinum	55/294	35/208	0.91 (0.57-1.44)	0.679	46/265	44/237	1.08 (0.69-1.69)	0.748	46/266	44/236	1.09 (0.69-1.70)	0.722
Others	17/294	9/208	0.74 (0.32-1.68)	0.467	11/265	15/237	1.50 (0.67-3.34)	0.320	11/266	15/236	1.52 (0.68-3.38)	0.307
Clinical stages												
I+II+4s	71/294	45/208	0.90 (0.59-1.36)	0.613	66/265	50/237	0.85 (0.56-1.27)	0.421	66/266	50/236	0.85 (0.57-1.28)	0.444
III+IV	89/294	53/208	0.84 (0.57-1.24)	0.385	59/265	83/237	**1.57 (1.07-2.29)**	**0.021**	59/266	83/236	**1.58 (1.08-2.31)**	**0.019**

AOR, adjusted odds ratio; CI, confidence interval.
[a] Adjusted for age and gender, omitting the corresponding stratification factor.

Supplementary Table 4. Stratification analysis of *MEG3* polymorphisms with neuroblastoma susceptibility (Henan province).

Variables	rs7158663 (cases/controls)		AOR (95% CI) [a]	*P* [a]	rs4081134 (cases/controls)		AOR (95% CI) [a]	*P* [a]	Risk genotypes (cases/controls)		AOR (95% CI) [a]	*P* [a]
	GG	AG/AA			GG	AG/AA			0-1	2		
Age, month												
≤18	11/32	12/40	0.85 (0.33-2.19)	0.736	15/44	8/28	0.79 (0.29-2.13)	0.642	15/44	8/28	0.79 (0.29-2.13)	0.642
>18	54/107	41/142	0.78 (0.48-1.27)	0.318	54/134	41/75	1.36 (0.83-2.24)	0.223	54/134	41/75	1.36 (0.83-2.24)	0.223
Gender												
Females	28/48	26/61	0.73 (0.38-1.40)	0.341	30/69	24/40	1.38 (0.71-2.68)	0.348	30/69	24/40	1.38 (0.71-2.68)	0.348
Males	37/91	27/81	0.84 (0.47-1.51)	0.562	39/109	25/63	1.05 (0.58-1.91)	0.868	39/109	25/63	1.05 (0.58-1.91)	0.868
Sites of origin												
Adrenal gland	53/139	36/142	0.65 (0.40-1.06)	0.087	51/178	38/103	1.28 (0.78-2.08)	0.327	51/178	38/103	1.28 (0.78-2.08)	0.327
Mediastinum	8/139	11/142	1.30 (0.50-3.35)	0.590	12/178	7/103	0.99 (0.38-2.61)	0.986	12/178	7/103	0.99 (0.38-2.61)	0.986
Others	4/139	6/142	1.53 (0.42-5.64)	0.519	6/178	4/103	1.11 (0.30-4.05)	0.878	6/178	4/103	1.11 (0.30-4.05)	0.878
Clinical stages												
I+II+4s	24/139	23/142	0.90 (0.49-1.69)	0.750	27/178	20/103	1.28 (0.68-2.40)	0.444	27/178	20/103	1.28 (0.68-2.40)	0.444
III+IV	39/139	29/142	0.72 (0.42-1.23)	0.232	40/178	28/103	1.19 (0.69-2.05)	0.530	40/178	28/103	1.19 (0.69-2.05)	0.530

AOR, adjusted odds ratio; CI, confidence interval.
[a] Adjusted for age and gender, omitting the corresponding stratification factor.

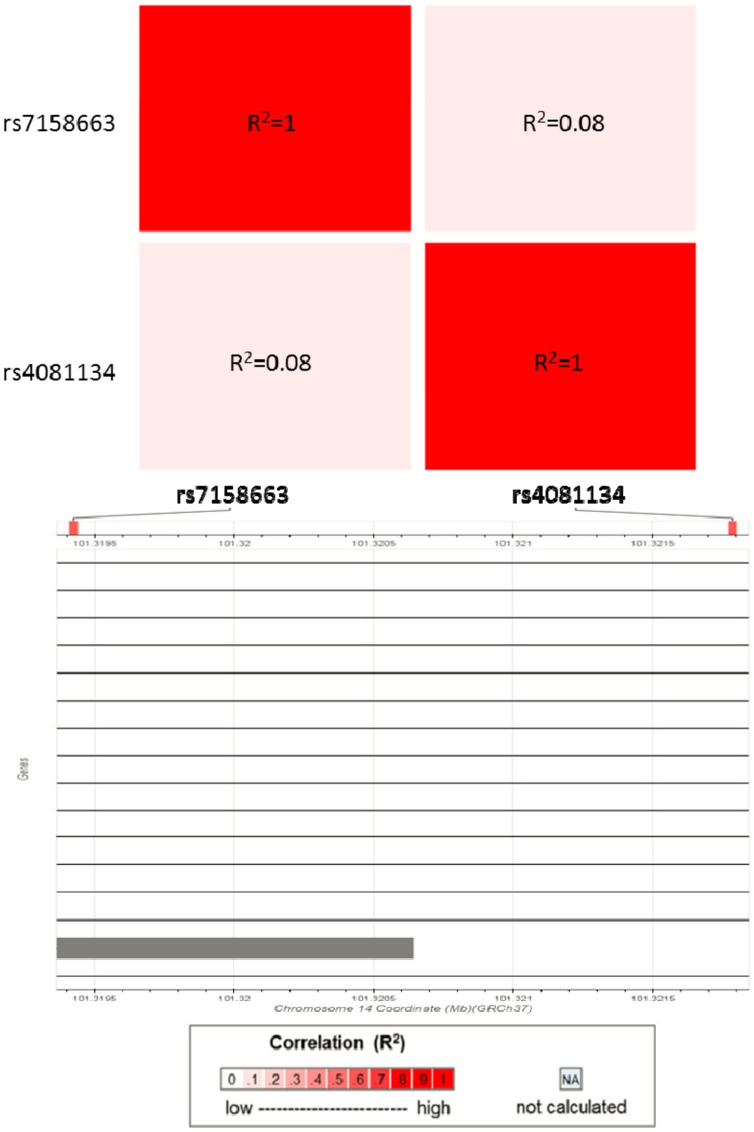

Supplementary Figure 1. Linkage disequilibrium analysis for the two selected *MEG3* polymorphisms in Han Chinese population consisted of CHB (Han Chinese in Beijing, China) and CHS (Southern Han Chinese) subjects.
